# Use of Point-of-Care Ultrasound and Focus-Assessed Transthoracic Echocardiography to Diagnose Acute Right Heart Failure Due to Fat Emboli in a Parturient

**DOI:** 10.7759/cureus.28585

**Published:** 2022-08-30

**Authors:** Stephanie O Ibekwe, Varun Potluri, Raja Palvadi, Gavin T Best

**Affiliations:** 1 Anesthesiology, Baylor College of Medicine, Houston, USA

**Keywords:** multidisciplinary care, fate, point of care ultrasound, fat embolism syndrome, pulmonary hypertension, fat embolism, right heart failure

## Abstract

Fat embolism syndrome (FES), causing right heart dysfunction, is a rare disease that is often difficult to diagnose with imaging modalities such as computed tomography (CT). FES is the clinical presentation that follows the entry of fat globules into the systemic circulation, which typically results in respiratory failure, scattered petechiae, cardiovascular collapse, and neurological sequelae. It is mostly observed in the cases of orthopedic trauma but may occur in any circumstance where fat can enter the circulatory system. In this case report, the authors describe an atypical presentation of FES in a 24-week parturient. The use of bedside point-of-care ultrasonography (POCUS) and the focus-assessed transthoracic echocardiography (FATE) protocol aided in the prompt diagnosis of right heart failure and helped to confirm the diagnosis of FES with more advanced imaging technology.

## Introduction

While fat emboli are often subclinical and undiagnosed, they can precipitate acute right heart failure [1]. This case presents several anesthetic and surgical challenges, particularly in the context of the physiologic changes during pregnancy with a co-existing viable fetus. A strong multidisciplinary approach is critical to preoperative optimization. It is equally important to maintain a high index of suspicion for fat emboli in susceptible patients. Point-of-care ultrasound (POCUS) and the focus-assessed transthoracic echocardiography (FATE) protocol are highly effective tools for cardiopulmonary perioperative assessment and management [2,3]. When combined with invasive monitoring, these non-invasive methods of assessment can help guide perioperative care.

This article was previously presented as a meeting abstract at the 2022 Society of Cardiovascular Anesthesiologists' Annual Scientific Meeting on May 14, 2022.

## Case presentation

A 25-year-old, American Society of Anesthesiologists (ASA) 2, G1P0 woman, with no reported chronic medical problems presented as a restrained passenger in a motor vehicle collision to the emergency room (ER) at 24 weeks of gestation. Her surgical history was significant for previous breast implantation and cosmetic fat transfer several years prior to presentation. She was hemodynamically stable, and her physical exam was significant for left ankle deformity, closed left mid-shaft tibia fracture, and left tibial plateau fracture. Fetal heart tones, ultrasound, computed tomography (CT) scan of the chest, abdomen, and pelvis, and chest x-ray were unremarkable for acute abnormality.

Orthopedic surgery performed a closed reduction of the ankle and admitted the patient for observation, planned open reduction, internal fixation (ORIF), and intramedullary nailing (IMN).

On hospital day one, the patient became tachycardic and hypoxemic with saturated pulse oximetry readings in the low 90s. Oxygen saturation improved with 2 liters of oxygen via nasal cannula. She continued to have increased oxygen requirements over the next 24 hours and became febrile to 102°F. The patient complained of tiredness; however, she had no chest pain, no orthopnea, or cough. The differential diagnosis included atelectasis, pneumonia, and hypoventilation due to opioid administration. Pulmonary embolism, fat embolism, and amniotic fluid embolism were less likely. A CT pulmonary angiography (CTPA) was performed and was negative for pulmonary emboli or thoracic abnormality. The patient was negative for coronavirus disease 2019 (COVID-19) and influenza types A and B. She also had negative blood and urine cultures.

**Figure 1:**
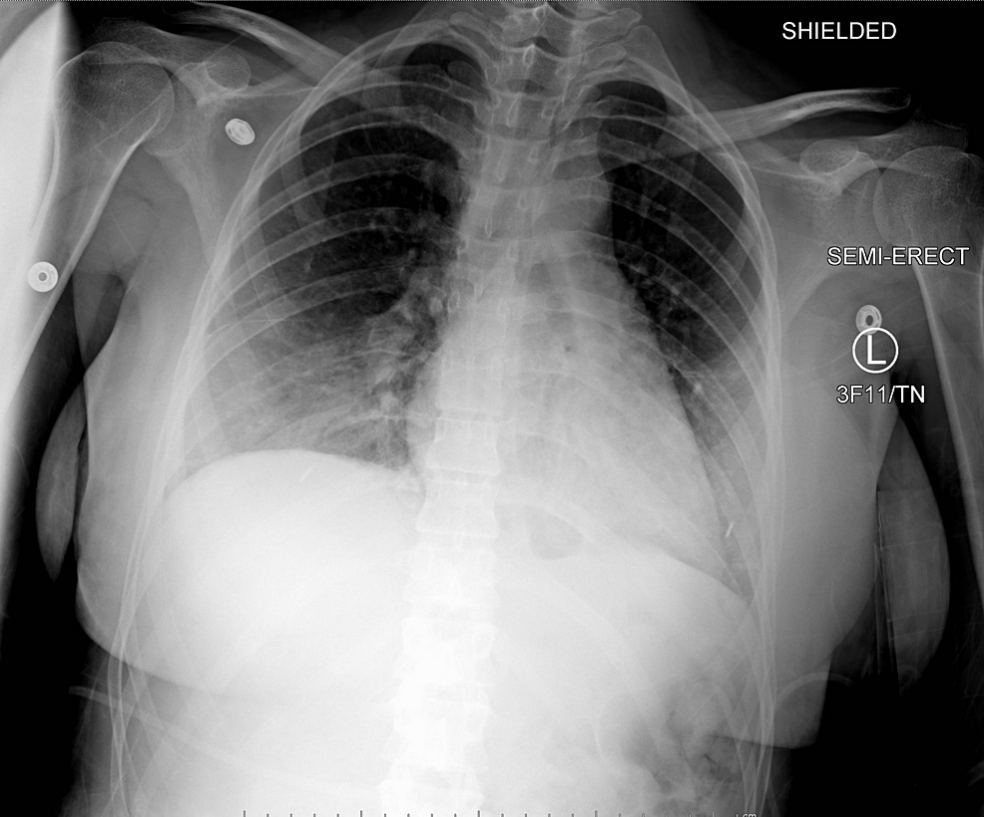
Chest radiograph on day of admission L: left

**Figure 2:**
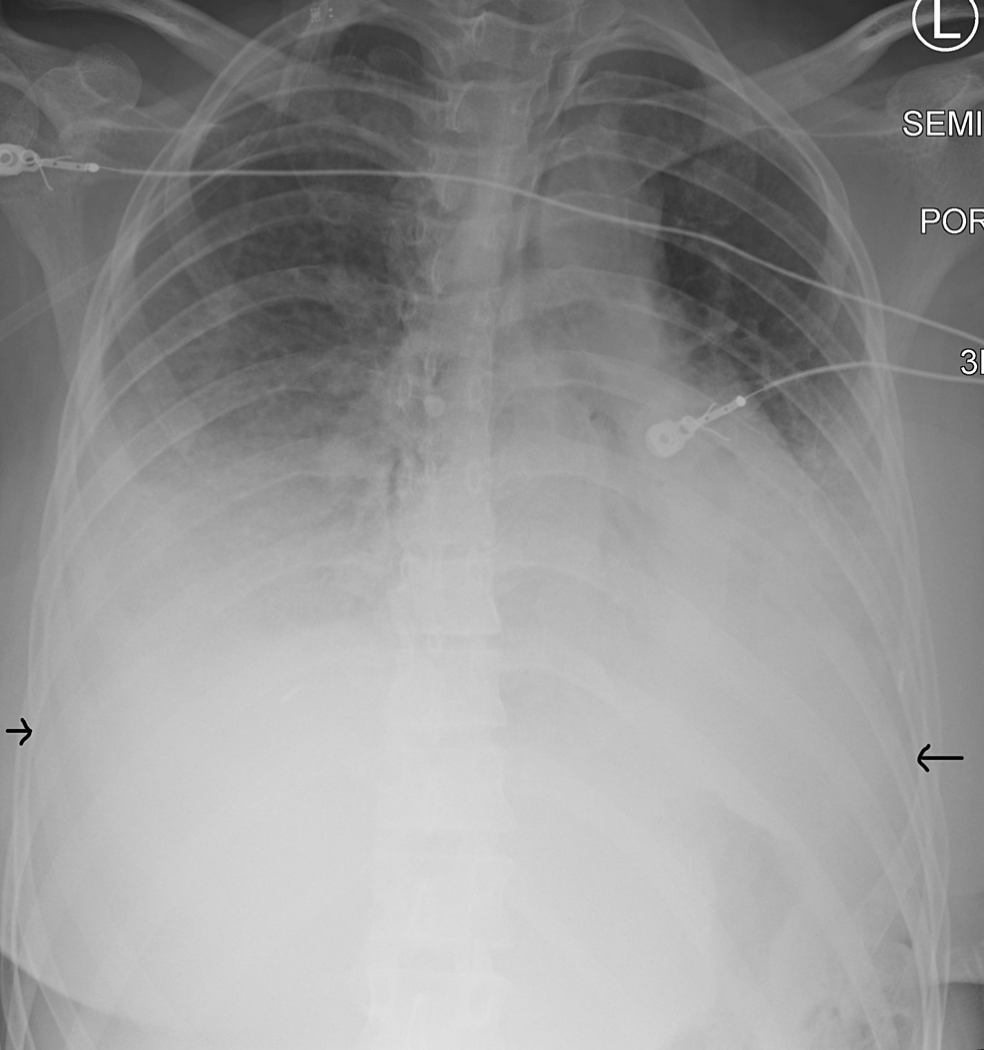
Chest radiograph on hospital day two with worsening of bibasilar hazy opacities L: left

A repeat chest x-ray was significant for worsening bibasilar hazy opacities consistent with mucous plugging vs. pulmonary edema vs. aspiration (Figure 1 and Figure 2). Arterial blood gas readings were as follows: pH 7.44, partial pressure of carbon dioxide (PCO2) 33, arterial oxygen pressure (PaO2) 112 on 10 liters of oxygen non-rebreather mask.

On hospital day three, the patient continued to have increased oxygen requirements, fever, and complaints of fatigue. The obstetric anesthesiology team performed a bedside POCUS exam of the lungs and heart using the FATE protocol.

Findings included bilateral lower lobe atelectasis, small pleural effusions, and B-lines over the anterior and posterior chest. FATE was significant for severe right ventricular dilation and reduced function, and the inferior vena cava was plethoric with less than 50% variability with respirations. These findings were consistent with acute versus chronic pulmonary embolism causing acute right heart failure.

**Figure 3:**
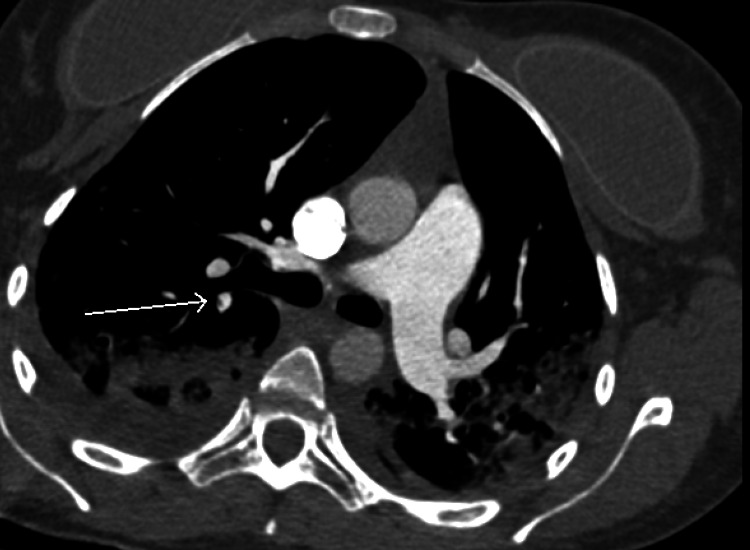
CT chest pulmonary embolism protocol demonstrating fat density branching filling defect within right upper lobe segmental pulmonary artery with new diffuse airspace disease including ground-glass opacities and interlobular septal thickening

**Figure 4:**
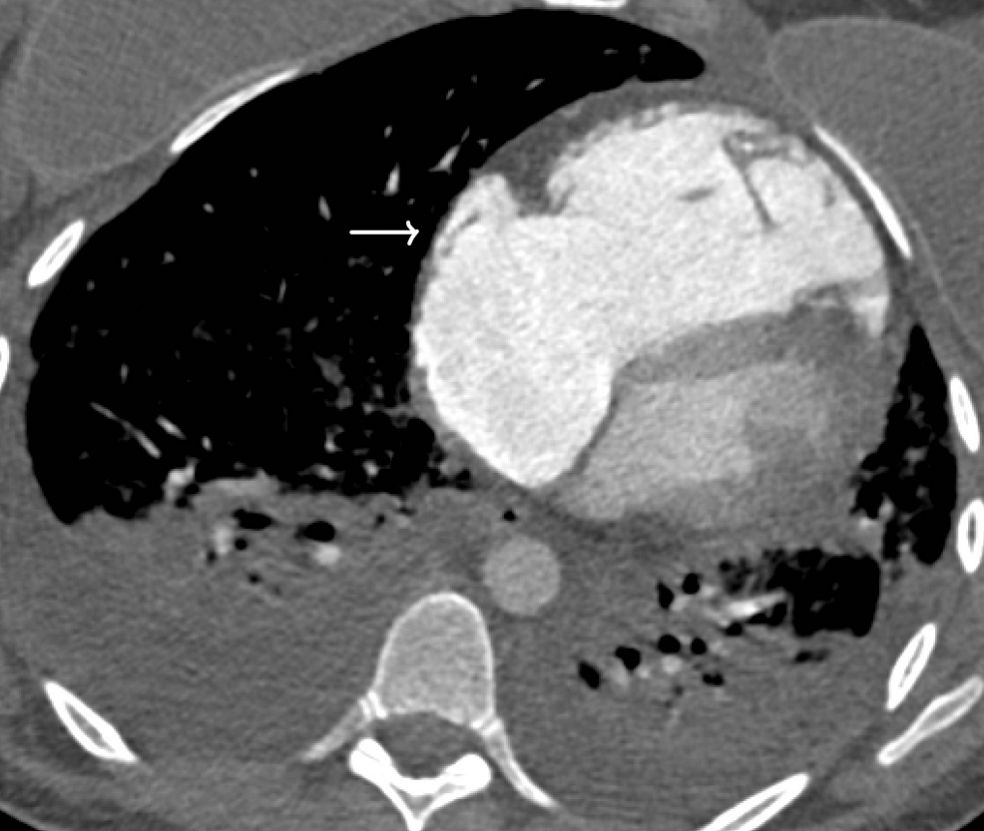
Severely dilated RV suggesting significant RV strain obtained on hospital day three RV: right ventricle

A repeat chest CT was significant for a fat density branching filling defect within a right upper lobe segmental pulmonary artery, and Hounsfield units of embolism consistent with fat embolism, not thrombus. CT findings were consistent with right heart strain. New diffuse airspace disease with micro-nodules, ground-glass opacities, and interlobular septal thickening were all noted and consistent with fat embolism syndrome (Figure 3 and Figure 4).

**Figure 5:**
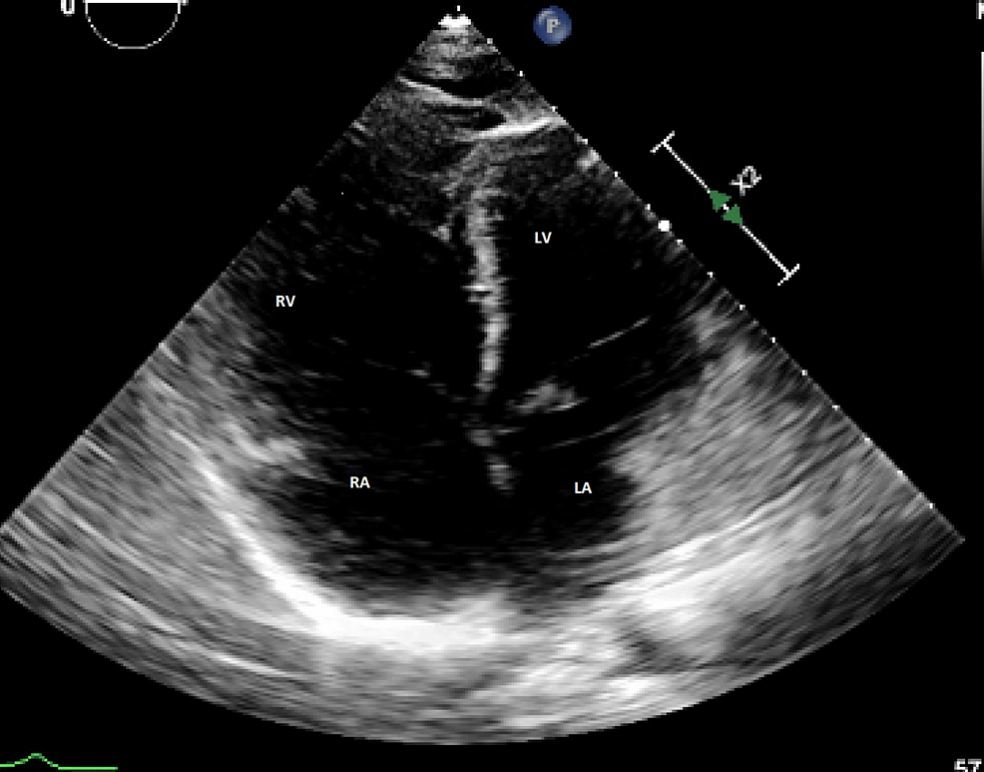
Transthoracic echocardiogram with apical four-view with severely dilated right ventricle and severely reduced right ventricular global systolic function and flattening of intraventricular septum seen during systole RV: right ventricle; RA: right atrium; LV: left ventricle; LA: left atrium

**Figure 6:**
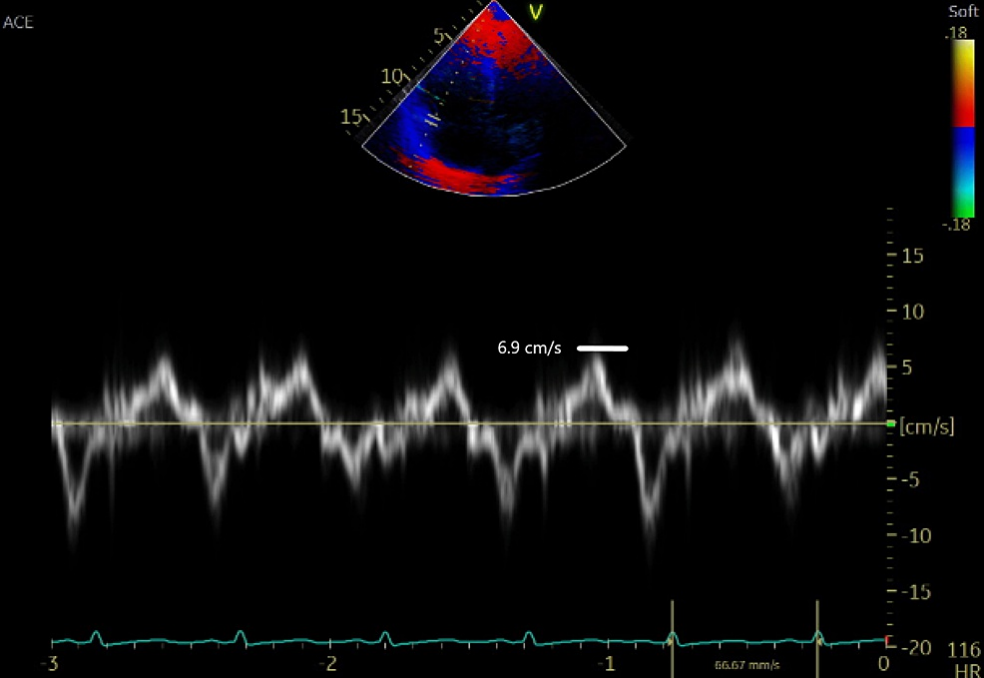
Right ventricular S’ reduced at 6.9 cm/s

**Figure 7:**
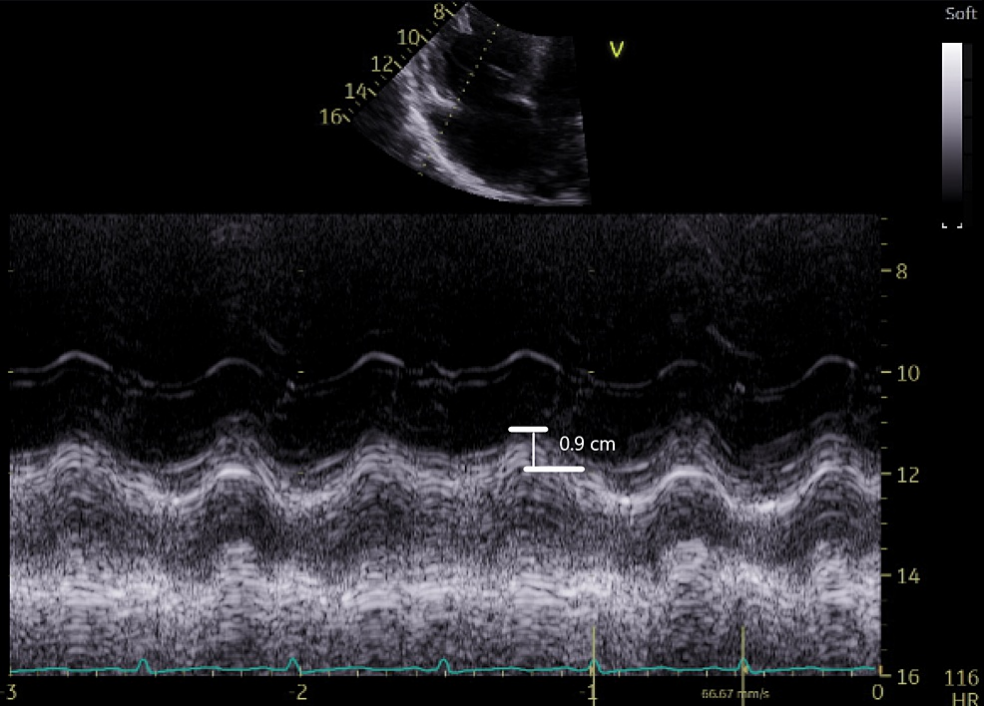
Right ventricular TAPSE 0.9 cm, consistent with severely depressed right ventricular systolic function TAPSE: tricuspid annular plane systolic excursion

The cardiology team performed a transthoracic echocardiogram (TTE) with the following findings: small left ventricular cavity, mild concentric left ventricular hypertrophy, and normal left ventricular systolic function with an ejection fraction of 65-69%. The flattened interventricular septum in systole was consistent with right ventricular pressure overload (Figure 5). Severe right ventricular dilation and severely reduced right ventricular global systolic function were observed. Tricuspid annular plane systolic excursion (TAPSE) was 0.9 cm and right ventricular S' was 6.9 cm/s (Figure 6 and Figure 7). Pulmonary artery systolic pressure (PASP) was estimated to be 44 mmHg assuming right atrial pressures were greater than 15 mmHg. She had elevated brain natriuretic peptide (BNP) levels at 699 pg/ml.

The patient was diagnosed with fat embolism syndrome. Supportive treatment including supplemental oxygen, continuous pulse oximeter monitoring, continuous fetal heart monitoring, and gentle diuresis with 10 mg of intravenous furosemide was initiated. Over several days, oxygen requirements waned, no inotropes were required to maintain normal mean arterial pressures, and fetal heart tones remained reassuring.

On hospital day 11, there was a multidisciplinary meeting with all the care teams to discuss the perioperative management of the patient. The patient's right heart failure, fat embolism causing respiratory depression, her periviable pregnancy, and the possibility of repeat fat embolism with operative manipulation were all considered during the discussion. Since this patient had a closed fracture, non-operative management of the fracture was an option, though the patient would likely suffer from long-term arthritic leg changes. When presented with this option, the patient preferred surgical management. This had to occur within two weeks of the injury for optimal outcomes.

Cardiology was consulted for right heart catheterization (RHC) and pulmonary artery catheter (PAC) placement, preoperatively. On hospital day 12, RHC revealed that pulmonary artery systolic pressure and wedge pressures were within normal limits, and CVP was 9. Additionally, BNP levels had declined to 452 pg/ml. The cardiology department assessed that the patient required no additional optimization, and that right ventricle pressure overload was improving.

On hospital day 14, the patient underwent ORIF and IMN of the left lower extremity fractures. Consultants were available from maternal-fetal medicine, neonatal medicine, cardiothoracic surgery, and perfusion for emergent delivery of the fetus and emergent management of repeat fat embolism should it recur. The planned anesthetic technique for this patient was neuraxial anesthesia.

A pre-procedural arterial line and standard ASA monitors were placed before combined spinal-epidural placement. The PAC monitored cardiac output continuously throughout the CSE placement and dosing. The anesthesiology team administered 10 micrograms of intrathecal fentanyl and threaded the epidural catheter into the epidural space. After noting a negative test dose, the team administered preservative-free 0.5% bupivacaine followed by 2% lidocaine to achieve an anesthetic level of T6.

The team administered a norepinephrine infusion at 2 mcg/minute for mild hypotension after block placement. Medications for anesthetic maintenance were low-dose norepinephrine, dexmedetomidine infusion titrated from 0.3 to 0.5 mcg/kg/minute, and 25 mcg boluses of fentanyl. The patient received a total of 40 mcg of dexmedetomidine, 4 mg of morphine, 2 mg of midazolam, and 200 mcg of fentanyl during the case.

The patient had an uncomplicated surgery and transferred to the ICU. Repeat TTE postoperatively showed moderate right ventricular dilation and moderately reduced right ventricular global systolic function with a TAPSE of 1.2 cm. The estimated PASP was estimated to be 25 mmHg. She was discharged home on hospital day 21.

## Discussion

Physiologic changes in the pulmonary and hematologic systems during pregnancy

There are several expected physiologic changes during pregnancy. The changes that most affect anesthetic management occur in the cardiovascular, hematologic, pulmonic, and gastroenterological systems. 

Pregnancy is a known hypercoagulable state with as much as a tenfold increased risk of deep venous thrombosis and pulmonary embolus [4]. There are significant increases in fibrinogen levels and von Willebrand Factor, Factors VII, VIII, and IX by 200% [5]. Anticoagulants like protein S are decreased, further contributing to the risk of thrombotic events. Due to these changes, most prothrombotic tests (i.e., D-dimer) have unreliable accuracy in evaluating pulmonary embolism in this patient population [5]. Activated partial thromboplastin clotting time (aPTT) and prothrombin time (PT) are typically shortened and less accurate toward the end of the third trimester [5].

The pulmonary system undergoes many changes during pregnancy. The chest wall relaxes and flares, the diaphragm is pushed upward by an enlarging uterus, and minute ventilation increases by over 50% by the third trimester. This increase in minute ventilation is almost entirely due to increased tidal volume since the respiratory rate stays roughly stable throughout pregnancy. While it is true that maternal oxygen demand is higher during pregnancy (due to increased cardiac and renal oxygen demand as well as metabolism by the fetus), an increase in ventilation is often more than what is required to compensate for this increase in demand. This is primarily due to progesterone, which directly stimulates the respiratory drive. This increased ventilation decreases pCO2, causing a respiratory alkalosis that is compensated partially by bicarbonate excretion and mild metabolic acidosis (Table 1) [6,7,8]. 

**Table 1:** Pregnant vs non-pregnant laboratory parameters PaO2: partial pressure of oxygen in arterial blood; PaCO2: partial pressure of carbon dioxide in arterial blood; HCO3: bicarbonate

	Nonpregnant	Pregnant (3^rd^ trimester)
Arterial pH	7.40	7.43 (7.40-7.45)
PaO_2_(mmHg)	93	101-106
PaCO_2_ (mmHg)	37-40	26-32
Serum HCO_3_ (mEq/L)	23	17-20
Hemoglobin (g/dL)	12-16	10-15
White blood cell count (x10^4^/ µL)	4-11	5-12
Fibrinogen (mg/dL)	150-400	>400
Albumin (g/L)	37-48	23-38
Creatinine (µmol/L)	73	64

Fat embolism 

Ultimately, this patient had a massive fat embolism in the pulmonary circulation. Fat embolism syndrome (FES) is the clinical presentation that follows the entry of fat globules into the systemic circulation, which typically results in respiratory failure, scattered petechiae, cardiovascular collapse, and neurological sequelae (altered mental status, focal deficits, and seizures). It is most commonly observed in the cases of orthopedic trauma but may occur in any circumstance where fat can enter the circulatory system. Kainoh et al. showed that long bone fractures, open fractures, the presence of multiple fractures, and a delay in fracture reduction are all risk factors for FES [9].

Fat embolism is typically a clinical diagnosis. Chest x-rays are typically normal in patients with FES. CTPA in these individuals typically shows bilateral ground-glass opacities, thickening of the interlobular septa, and centrilobular nodular opacities. FES may have no filling defects like those seen in large classic thromboembolic PE. When they are visible on CTPA, the disease is severe, as was the case in this patient [10]. Brain MRI ordered in patients with altered mental status (AMS) or focal deficits will show a "star field" pattern in the parenchyma that correlates to hyperlucent punctate lesions [10].

No laboratory test will reliably confirm FES. Invasive tests are similarly not warranted [11]. While there may be observance of fat globules in the sputum or secretions of patients with fat embolism, studies have shown no reliable correlation that makes this sign clinically significant [1,11,12].

As mentioned in this patient's case, the differential diagnosis of the patient with FES includes pulmonary embolism, amniotic fluid embolism, tumor embolism, air embolism, pneumonia, pulmonary contusion, and foreign body embolism. In this pregnant trauma patient, amniotic fluid embolism was high on the differential. However, this pathology involves an anaphylactoid serum reaction to fetal tissues in the maternal plasma. While it presents with cardiovascular collapse, respiratory failure, and seizures, it won’t reflect on CTPA [11]. Pulmonary embolism would cause this level of right heart failure as in the current patient; however, a thrombotic embolism can be distinguished from fat by measuring the Hounsfield units [10].

Treatment for this condition remains supportive. Trials of steroid and heparin administration have not consistently shown improved outcomes; both may cause adverse effects [13].

TTE/ POCUS right heart failure findings

The patient experienced acute right heart failure secondary to fat embolism causing acute pulmonary hypertension. FATE protocol helped assess right heart function.

Due to its mobile and facile nature, bedside POCUS is becoming more prevalent [2]. The FATE protocol is an abbreviated TTE protocol used to perform a quick, real-time assessment of cardiac function [3]. The basic FATE views require acquiring five views: subcostal view, apical four-chamber view, parasternal long-axis view, and pleural scanning [3].

Because of the right ventricle (RV) geometry and the complex three-dimensional (3D) shape, the measurement of RV function (RVF) is a challenge. Visual examination is the most used method to quantify RVF [14]. Several echocardiographic RVF parameters have been established and validated; however, each has significant limitations [14].

Our patient was diagnosed with acute right heart failure secondary to pulmonary hypertension using the FATE protocol. Initial TAPSE measured 0.9 cm. TAPSE is a rapid and reproducible parameter as it is a surrogate of the longitudinal fibers' function [15]. It measures the tucking effect of the apex on the tricuspid annulus [15]. Although TAPSE is an angle-dependent measurement, its measurement is not significantly affected by loading conditions. Longitudinal displacement of 1.7 cm or less indicates poor RV function and prognosis [15].

Estimating pulmonary hypertension is integral to evaluating a patient with suspected RVF. PASP is estimated non-invasively in the absence of pulmonary stenosis by measuring the peak velocity of tricuspid regurgitation, applying the simplified Bernoulli equation, and adding that value to the estimated right atrial pressure [15].

**Figure 8:**
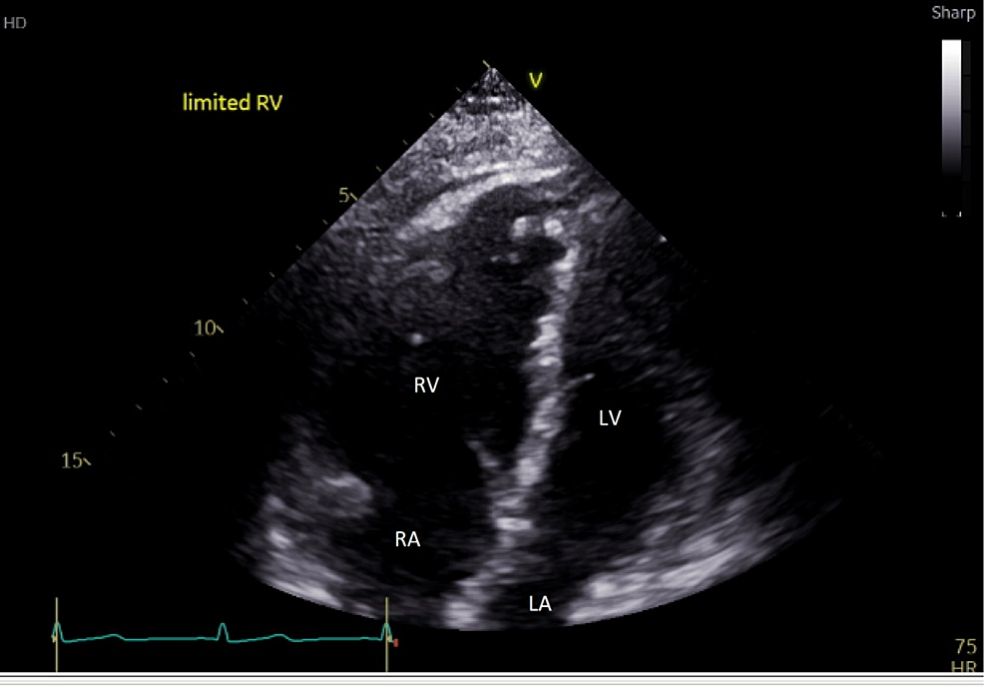
Severely dilated RV, mild to moderately dilated RA, septal flattening and bowing into LV during systole RV: right ventricle; RA: right atrium; LV: left ventricle; LA: left atrium

In symptomatic patients, a peak tricuspid regurgitation velocity >2.8 m/s is consistent with the presence of significant pulmonary hypertension. Other parameters consistent with significant pulmonary hypertension include the following: distended inferior vena cava (IVC) >21 mm with decreased inspiratory collapse [16], dilated RV, flattening of the interventricular septum, the D sign during systole (Figure 8) [17], pulmonary artery diameter > 25 mm, dilated right atrium (RA), and short pulmonary valve acceleration time. Flattening of the septum during diastole is significant for volume overload [17].

In our case, POCUS examination B-lines were present in the lung fields. B-lines are transient, hyperechoic vertical lines that extend from the pleura to the bottom of the screen at a depth of 16cm [18]. The presence of three or more B-lines is a pathologic finding [13]. Focal B-lines may suggest pneumonia. In contrast, diffuse B-lines in three or more zones on both sides of the chest suggest a diffuse alveolar interstitial syndrome such as pulmonary edema or acute respiratory distress syndrome [18].

Perioperative management of acute pulmonary hypertension and right heart dysfunction for non-cardiac surgery

The patient experienced acute right heart failure secondary to fat embolism, causing acute pulmonary hypertension. The most recent guidelines from the American Heart Association/American College of Cardiology (AHA/ACC) Foundation Practice Guideline for non-cardiac surgery lists pulmonary hypertension as an independent risk factor for postoperative complications [19]. 

Steppan et al. published guidelines on the perioperative management of patients with pulmonary hypertension [20]. In the presented case, the multidisciplinary team approach includes the anesthesiologist, maternal-fetal medicine specialist, pulmonary hypertension specialist, and surgeon. Due to the complex nature of the care for patients with pulmonary hypertension, goals should include avoiding emergent surgery and considering all risks and benefits of any surgical intervention planned [20].

Anesthetic goals are to avoid right heart ischemia and failure, significant increases in pulmonary artery pressure, and acute changes in right ventricular cardiac output. Avoiding systemic hypotension, myocardial depression, acute pulmonary artery pressure elevations, inadequate pain control, and respiratory depression supports these anesthetic goals [20].

Considering the need for maintaining good control over hemodynamics, invasive monitoring is sapient in patients with pulmonary hypertension and right heart dysfunction during the onset and maintenance of anesthesia. In addition to standard ASA monitors (ECG, pulse oximetry, and temperature), a preinduction arterial catheter may be helpful to monitor the systemic blood pressure more closely with the induction of general anesthesia or regional block. PAC monitoring helps assess the patient's cardiac output, mixed venous saturation, central venous pressure monitoring, pulmonary vascular resistance (PVR), and pulmonary capillary wedge pressure. Transesophageal echocardiography (TEE) can be inserted in intubated patients to evaluate pulmonary arterial pressure and right ventricular function and size and assess fluid and inotrope administration intra-operatively. 

## Conclusions

This case highlights the importance of having an increased index of suspicion for FES in patients at high risk for the syndrome. In this case, a recent trauma resulting in a long bone fracture, a surgical history of fat transfer, and acute signs of cardiac failure put FES on the differential diagnosis despite initial negative CT imaging results. POCUS and the FATE protocol aided in the prompt diagnosis of cardiovascular dysfunction in this parturient, ultimately leading to the correct diagnosis and treatment. The patient’s medical complexity necessitated a strong multidisciplinary approach for perioperative optimization and successful surgical intervention. With thorough perioperative planning and a well-executed surgical procedure, this patient, had a good outcome.
